# Supplementary material for: Desmodium volatiles in ‘push-pull’ cropping systems and protection against the fall armyworm, Spodoptera frugiperda
Source: eLife. 2025 Oct 9;13:RP100981. doi: 10.7554/eLife.100981 (PMC12510683; doi:10.7554/eLife.100981)
Supplement: MDAR checklist [file elife-100981-mdarchecklist1.pdf]

Materials Design Analysis Reporting (MDAR) Checklist for Authors

The MDAR framework establishes a minimum set of requirements in transparent reporting mainly applicable to studies in the life sciences.

eLife asks authors to **provide detailed information within their article** to facilitate the interpretation and replication of their work. Authors can also upload supporting materials to comply with relevant reporting guidelines for health-related research (see EQUATOR Network), life science research (see the BioSharing Information Resource), or animal research (see the ARRIVE Guidelines and the STRANGE Framework; for details, see *eLife*'s Journal Policies). Where applicable, authors should refer to any relevant reporting standards materials in this form.

For all that apply, please note **where in the article** the information is provided. Please note that we also collect information about data availability and ethics in the submission form.

Materials:

Newly created materials	Indicate where provided: section/figure legend	N/A
The manuscript includes a dedicated "materials availability statement" providing transparent disclosure about availability of newly created materials including details on how materials can be accessed and describing any restrictions on access.		No creation of new materials

Antibodies	Indicate where provided: section/figure legend	N/A
For commercial reagents, provide supplier name, catalogue number and RRID, if available.		No use of antibodies

DNA and RNA sequences	Indicate where provided: section/figure legend	N/A
Short novel DNA or RNA including primers, probes: Sequences should be included or deposited in a public repository.		No sequencing of DNA / RNA

Cell materials	Indicate where provided: section/figure legend	N/A
Cell lines: Provide species information, strain. Provide accession number in repository OR supplier name, catalog number, clone number, OR RRID.		No use of cell materials

**Cell materials**	**Indicate where provided: section/figure legend**	**N/A**
Primary cultures: Provide species, strain, sex of origin, genetic modification status.		No use of cell materials

**Experimental animals**	**Indicate where provided: section/figure legend**	**N/A**
Laboratory animals or Model organisms: Provide species, strain, sex, age, genetic modification status. Provide accession number in repository OR supplier name, catalog number, clone number, OR RRID.	Details are provided in the Methods section of the oviposition, dual-choice and no-choice bioassay. In short, fall armyworm moths (*Spodoptera frugiperda*) were reared from wild individuals collected in the counties Siaya, Kisumu, Migori, and Vihiga. Sex was determined visually at the adult stage. Individuals used in experiments were 4–5-day post-emergence.	
Animal observed in or captured from the field: Provide species, sex, and age where possible.	Details are provided in the Methods section of the oviposition, dual-choice and no-choice bioassay. Wild *Spodoptera frugiperda* adults were collected to establish laboratory colonies. Sex was determined visually at the adult stage. Age at the time of collection could not be determined with certainty.	

**Plants and microbes**	**Indicate where provided: section/figure legend**	**N/A**
Plants: provide species and strain, ecotype and cultivar where relevant, unique accession number if available, and source (including location for collected wild specimens).	Details, including GPS coordinates of the headspace sampling are provided in the Methods section. *Desmodium intortum* and *Desmodium incanum* were visually identified in farmers' fields.	
Microbes: provide species and strain, unique accession number if available, and source.		No use of microbes

**Human research participants**	**Indicate where provided: section/figure legend) or state if these demographics were not collected**	**N/A**
If collected and within the bounds of privacy constraints report on age, sex, gender and ethnicity for all study participants.		No involvement of human participants

Design:

Study protocol	Indicate where provided: section/figure legend	N/A
If the study protocol has been pre-registered, provide DOI. For clinical trials, provide the trial registration number OR cite DOI.		No pre-registration

Laboratory protocol	Indicate where provided: section/figure legend	N/A
Provide DOI OR other citation details if detailed step-by-step protocols are available.	Detailed descriptions of bioassay and sampling protocols are provided in the Methods section.	

Experimental study design (statistics details) *		
**For in vivo studies: State whether and how the following have been done**	**Indicate where provided: section/figure legend. If it could have been done, but was not, write "not done"**	**N/A**
Sample size determination		No in vivo study
Randomisation		No in vivo study
Blinding		No in vivo study
Inclusion/exclusion criteria		No in vivo study

Sample definition and in-laboratory replication	Indicate where provided: section/figure legend	N/A
State number of times the experiment was replicated in the laboratory.	Replication numbers for the different experiments are provided in the Methods section.	
Define whether data describe technical or biological replicates.	Details are provided in the Methods section. Headspace sampling, the oviposition bioassay, and the dual-choice bioassay were conducted with biological replicates. In the no-choice bioassay, biological replicates were included; however, only the moth was replicated, while the plants were exchanged less frequently.	

Ethics	Indicate where provided: section/submission form	N/A
Studies involving human participants: State details of authority granting ethics approval (IRB or equivalent committee(s), provide reference number for approval.		No human participants involved
Studies involving experimental animals: State details of authority granting ethics approval (IRB or equivalent committee(s), provide reference number for approval.		No permit required for lower organisms in Kenya.
**Ethics**	**Indicate where provided: section/submission form**	**N/A**
Studies involving specimen and field samples: State if relevant permits obtained, provide details of authority approving study; if none were required, explain why.		No permit required for lower organisms in Kenya.

Dual Use Research of Concern (DURC)	Indicate where provided: section/submission form	N/A
If study is subject to dual use research of concern regulations, state the authority granting approval and reference number for the regulatory approval.		DURC criteria are not met

Analysis:

Attrition	Indicate where provided: section/figure legend	N/A
Describe whether exclusion criteria were pre-established. Report if sample or data points were omitted from analysis. If yes, report if this was due to attrition or intentional exclusion and provide justification.	Details are provided in the Methods section. In short, non-decisive repetitions were excluded from the dual-choice and no-choice bioassays according to pre-established criteria. Several headspace sampling repetitions were excluded due to sampling deviations, with these decisions taken after sampling but before statistical analysis.	

Statistics	Indicate where provided: section/figure legend	N/A
Describe statistical tests used and justify choice of tests.	Mixed models were used to account for fixed and random effects in the experimental design, with full formulas provided in the Methods section. This approach appropriately handles repeated measures and variation between biological replicates.	

**Data availability**	**Indicate where provided: section/submission form**	**N/A**
For newly created and reused datasets, the manuscript includes a data availability statement that provides details for access (or notes restrictions on access).	All raw datasets are publicly available on Zenodo, as referenced in the Methods section.	
When newly created datasets are publicly available, provide accession number in repository OR DOI and licensing details where available.	The raw data is available on Zenodo (https://doi.org/10.5281/zenodo.11633889)	
If reused data is publicly available provide accession number in repository OR DOI, OR URL, OR citation.		No data reused

**Code availability**	**Indicate where provided: section/figure legend**	**N/A**
For any computer code/software/mathematical algorithms essential for replicating the main findings of the study, whether newly generated or re-used, the manuscript includes a data availability statement that provides details for access or notes restrictions.	All raw code is publicly available on GitHub, as referenced in the Methods section.	
Where newly generated code is publicly available, provide accession number in repository, OR DOI OR URL and licensing details where available. State any restrictions on code availability or accessibility.	The code for statistics is available on GitHub (https://github.com/DariaMOdermatt/DesmodiumVolatilesinPush-Pull)	
If reused code is publicly available provide accession number in repository OR DOI OR URL, OR citation.		No public code reused

Reporting:

The MDAR framework recommends adoption of discipline-specific guidelines, established and endorsed through community initiatives.

**Adherence to community standards**	**Indicate where provided: section/figure legend**	**N/A**
State if relevant guidelines (e.g., ICMJE, MIBBI, ARRIVE, STRANGE) have been followed, and whether a checklist (e.g., CONSORT, PRISMA, ARRIVE) is provided with the manuscript.		No use of specific guidelines

* We provide the following guidance regarding transparent reporting and statistics; we also refer authors to Ten common statistical mistakes to watch out for when writing or reviewing a manuscript.

Sample-size estimation

- You should state whether an appropriate sample size was computed when the study was being designed
- You should state the statistical method of sample size computation and any required assumptions
- If no explicit power analysis was used, you should describe how you decided what sample (replicate) size (number) to use

Replicates

- You should report how often each experiment was performed
- You should include a definition of biological versus technical replication
- The data obtained should be provided and sufficient information should be provided to indicate the number of independent biological and/or technical replicates
- If you encountered any outliers, you should describe how these were handled
- Criteria for exclusion/inclusion of data should be clearly stated
- High-throughput sequence data should be uploaded before submission, with a private link for reviewers provided (these are available from both GEO and ArrayExpress)

Statistical reporting

- Statistical analysis methods should be described and justified
- Raw data should be presented in figures whenever informative to do so (typically when N per group is less than 10)
- For each experiment, you should identify the statistical tests used, exact values of N, definitions of center, methods of multiple test correction, and dispersion and precision measures (e.g., mean, median, SD, SEM, confidence intervals; and, for the major substantive results, a measure of effect size (e.g., Pearson's r, Cohen's d)
- Report exact p-values wherever possible alongside the summary statistics and 95% confidence intervals. These should be reported for all key questions and not only when the p-value is less than 0.05.

Group allocation

- Indicate how samples were allocated into experimental groups (in the case of clinical studies, please specify allocation to treatment method); if randomization was used, please also state if restricted randomization was applied
- Indicate if masking was used during group allocation, data collection and/or data analysis
